# Antidiabetic Potential of Marine Brown Algae—a Mini Review

**DOI:** 10.1155/2020/1230218

**Published:** 2020-04-25

**Authors:** Thilina L. Gunathilaka, Kalpa Samarakoon, Pathmasiri Ranasinghe, L. Dinithi C. Peiris

**Affiliations:** ^1^Department of Zoology, Faculty of Applied Sciences, University of Sri Jayewardenepura, Nugegoda 10250, Sri Lanka; ^2^National Science and Technology Commission, Dudley Senanayake Mawatha, Colombo 8 00800, Sri Lanka; ^3^Industrial Technology Institute, Halbarawa Gardens, Malabe 10115, Sri Lanka

## Abstract

Marine algae are an important source of bioactive metabolites in drug development and nutraceuticals. Diabetes mellitus is a metabolic disorder and the third leading cause of death worldwide due to lifestyle changes associated with rapid urbanization. Due to the adverse side effects of currently available antidiabetic drugs, search for an effective natural-based antidiabetic drug is important to combat diabetes and its complications. Therefore, in lieu with herbal drug development, it is important to find the potential benefits of seaweeds for the management of type 2 diabetes as they are underexplored yet in Sri Lanka. Among the marine seaweeds, natural bioactive compounds are abundant in brown algae with potentials in application as active ingredients in drug leads and nutraceuticals. Bioactive secondary metabolites are derived from numerous biosynthetic pathways of marine algae which contribute to various chemical and biological properties. Phlorotannins present in marine brown algae exhibited antidiabetic activities through different mechanisms such as the inhibitory effect of enzyme targets mainly by inhibiting the enzymes such as *α*-amylase, *α*-glucosidase, angiotensin-converting enzymes (ACE), aldose reductase, dipeptidyl peptidase-4, and protein tyrosine phosphatase 1B (PTP 1B) enzyme. In addition, phlorotannins derived from brown algae have the ability to reduce diabetic complications. Hence, the present review focuses on the different antidiabetic mechanisms of secondary bioactive compounds present in marine brown algae.

## 1. Introduction

Diabetes mellitus (DM) is a metabolic disorder linked with chronic hyperglycaemia due to the relative or absolute deficiency in insulin hormone [1]. It is the third leading cause of death worldwide associated with major complications such as diabetic nephropathy, neuropathy, retinopathy leading to adult blindness, and amputations due to diabetic foot ulcers [2]. Though several pathogenic processes are involved with diabetes, most cases of diabetes can be categorized into two types depending on the etiology. Type 1 is defined as insulin-dependent DM, while type 2 is defined as noninsulin-dependent DM. Type 1 DM is associated with an absolute deficiency of insulin due to the autoimmune destruction of beta cells of the pancreas, which normally secrete insulin, whereas type 2 DM is associated with a relative deficiency of insulin due to the insulin resistance caused by an unhealthy diet, sedentary lifestyle, and obesity [3]. Both types of DM have different clinical and pathological features. However, type 2 DM is mostly prevalent among the world population, and it is associated with insulin resistance due to overproduction of glucose by the liver and underutilization of glucose by muscle and adipose tissues [4]. According to the recent WHO statistics, the world's diabetes population has increased up to 592 million by 2035 [5]. Among the two types of DM, type 1 DM is more common in the Northern European countries, whereas type 2 is most common in Africa and South Asian countries [6]. Hence, type 2 DM is mostly prevalent in Sri Lanka. In addition, more than 471 billion US dollars is spent annually worldwide for health care expenditure in diabetes patients and Sri Lankan government spend around 144 million US dollars annually to manage DM [7]. Therefore, DM not only affects the lives around world but also causes serious financial burden.

Owing to the pathogenesis of type 2 DM, insulin resistance is the main cause which leads to the development of type 2 DM. Insulin resistance is mostly developed in obese individuals than normal, and it can compensate with the overproduction of insulin by the *β* cells of the pancreas. Due to the overproduction of insulin, *β* cell functions will be impaired and will ultimately lead to chronic postprandial hyperglycaemia and fasting hyperglycaemia. Chronic hyperglycaemia can further diminish the function of *β* cells and enhance the status of insulin resistance [8]. In addition to chronic hyperglycaemia, type 2 DM is associated with dyslipidemia which affect the normal process of lipid metabolism [9]. Therefore, therapeutic strategies for type 2 DM should be developed to normalize the glucose metabolism and long-term complications.

Currently available therapeutic interventions for type 2 diabetes mellitus such as insulin administration and oral antidiabetic drugs have either limited efficacy or detrimental side effects [10]. Therefore, it is essential to keep searching for an effective drug that may benefit patients suffering from type 2 DM which leads towards the curing of these patients. So far, none of the drugs that are used to treat type 2 DM have full efficacy, nor have the scientific investigations yielded any potential drug for proper therapy. Therefore, there is an increase need in search for the new plant-based compounds with minimum or no side effects to the patients. Recourses from plant materials pose less chemical hazards and are proved to be an efficient application. Phytochemical compounds in plant extracts are known to contain a secondary metabolite that can be used effectively to manage various diseases including diabetes mellitus [11].

In addition to the medicinal plants, natural bioactive compounds are abundant in marine algae with potential active ingredients in the treatment of type 2 diabetes mellitus [12]. Therefore, in lieu with herbal drug development with minimum side effects and the high economic cost benefit, identification of chemical components and isolation of the active compounds in less utilized marine algae are of high importance. In particular, most of the brown algae are rich in important secondary metabolites such as phlorotannins which are reported to have an antidiabetic activity [13]. Therefore, the purpose of this review is to focus on the diverse antidiabetic mechanisms of brown algal compounds and their possible use in pharmaceutical industry.

## 2. Therapeutic Targets for Type 2 Diabetes Mellitus

Reduction of postprandial hyperglycaemia is one of the main therapeutic interventions to treat type 2 DM [14]. Postprandial hyperglycaemia can be reduced by inhibiting the carbohydrate-hydrolysing enzymes such as *α*-amylase and *α*-glucosidase. Alpha amylase and *α*-glucosidase are the important exo-acting glycoside hydrolase enzymes involved in the digestion of carbohydrates, and they act synergistically to digest starch in the human body. Alpha amylase breaks down the large insoluble starch by hydrolysing the alpha bonds, while *α*-glucosidase catalyses the end step of digestion of starch and disaccharides into glucose subunits. Therefore, the inhibition of such enzymes leads to the reduction of postprandial blood glucose level and these enzyme inhibitors act as a potential target for the development of antidiabetic drugs [15].

In addition to the carbohydrate-hydrolysing enzymes, the inhibition of enzymes such as aldose reductase, angiotensin-converting enzymes, dipeptidyl peptidase-4, and protein tyrosine phosphatase 1B can also be applied to the development therapeutic strategy to treat type 2 DM [16, 17]. Aldose reductase is a key metabolic enzyme involved in the polyol pathway which converts glucose into the sorbitol in the presence of NADPH as a cofactor. Hyperglycaemia in type 2 DM results in an overproduction of sorbitol through the polyol pathway. High intracellular accumulation of sorbitol leads to the development of cataract and diabetic neuropathy [18]. Therefore, inhibitors of aldose reductase can prevent the formation of sorbitol through the polyol pathway and reduce long-term diabetes complications.

**Figure 1:**
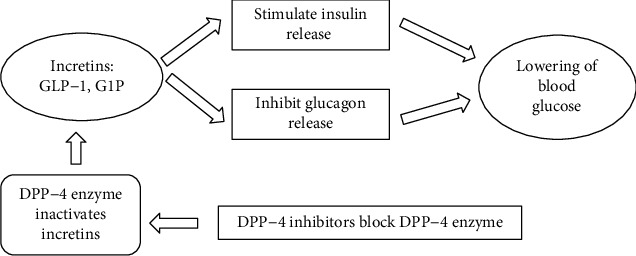
Action of DPP-4 inhibitors on glucose homeostasis.

The angiotensin-converting enzyme is involved with the renin-angiotensin-aldosterone system which converts angiotensin 1 into angiotensin II. Angiotensin II is a potent vasoconstrictor, and it stimulates the secretion of aldosterone by the adrenal cortex and increases sodium and water absorption. The activation of the renin-angiotensin-aldosterone system increased blood pressure, which causes microvascular and macrovascular complications in patients with type 2 DM [19]. Therefore, inhibitors of angiotensin-converting enzyme have the ability to lower the blood pressure, thus helping to diminish the long-term diabetic complications. Similarly, dipeptidyl peptidase-4 is an enzyme involved in glucose metabolism and helps to reduce incretin levels, such as glucose-like peptide-1 (GLP-1). GLP-1 is a gut hormone involved in the reduction of blood glucose level by stimulating insulin secretion during the hyperglycaemic conditions in patients with type 2 DM, whereas the effect of GLP-1 on insulin secretion progressively diminishes once the patient becomes euglycaemic [17]. Therefore, inhibitors of dipeptidyl peptidase-4 increase GLP-1 level which stimulates insulin secretion, thus helping to maintain hyperglycaemic conditions in patients with type 2 DM (Figure 1).

Protein tyrosine phosphatase 1B (PTP 1B) is another enzyme which is involved in the insulin signalling pathway and located on the cytoplasmic face of the endoplasmic reticulum. [20]. PTP 1B enzyme is a negative regulator of the tyrosine phosphorylation cascade which is directly involved with the insulin signalling pathway. The binding of insulin to insulin receptor causes structural changes in the receptor through the autophosphorylation of tyrosine residues within the cells, which subsequently activate the different pathways to increase glucose uptake to the cells. During this process, PTP 1B enzyme alters the insulin receptor phosphorylation and counteracts insulin signalling. Therefore, the inhibition of this enzyme leads to the reduction of the blood glucose level by increasing the insulin sensitivity; thus, protein tyrosine phosphatase 1B inhibitors can be used as a potential target for the treatment of type 2 DM [21].

Chronic hyperglycaemia in diabetic patients leads to the formation of advanced glycation end products which is associated with the pathogenesis of vascular complications in diabetes, renal failure, Alzheimer's disease, aging, and other chronic diseases. Formation of advanced glycation end products is a complex process, which involves the nonenzymatic reaction between the carbonyl group of a glucose molecule and free amino groups which leads to the formation of a nonstable Schiff base and Amadori products through reversible reactions. Further rearrangement of the Schiff base and Amadori products leads to the formation of advanced glycation end products through irreversible reactions [22]. Therefore, the natural compounds which inhibit the formation of advanced glycation end products can be used to suppress the diabetic complications associated with the accumulation of advanced glycation end products.

## 3. Bioactive Compounds Present in Marine Brown Algae

Marine macroalgae have been widely studied in the last few years due to the presence of human beneficial bioactive components. Marine seaweeds are a group of macroscopic and multicellular species living in the marine environment. Among them, macroalgae are large aquatic photosynthetic plants found in the coastal areas and they are visible to the naked eye. They have a high growth rate during the cultivation in sea water. Depending on the pigment they contain, macroalgae are classified as red algae (*Rhodophyta*), green algae (*Chlorophyta*), and brown algae (*Phaeophyta*) [23] Among the marine seaweeds, around 1500-2000 species of marine brown algae are available and most of the brown algal species are subjected to research purposes due to their commercial significance in drug leads and nutraceuticals. In particular, most important secondary bioactive metabolites such as phlorotannins, fucosterols, fucoidans, alginic acids, fucoxanthin, and phycocolloids have been extensively found in brown algae, which exhibit significant biological properties including antidiabetic, anti-inflammatory, cytotoxic, and antioxidant activities [24]. Here, we focus on the researches conducted worldwide to determine the antidiabetic activities of marine brown algae.

**Figure 2:**
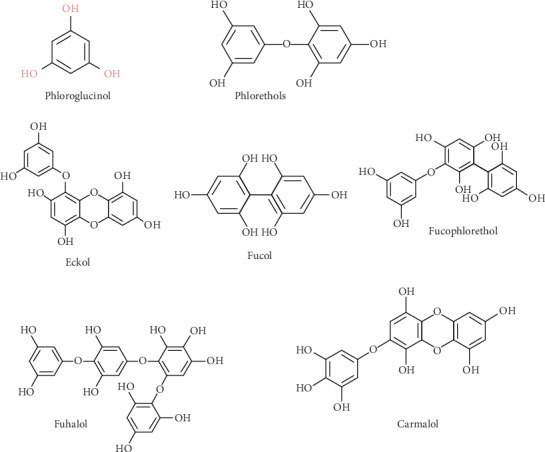
Chemical structure of phloroglucinol and groups of phlorotannins.

Phlorotannins are polyphenolic secondary metabolites derived from phloroglucinol and present mainly in marine brown algae. Phlorotannins are made up of repeating subunits of phloroglucinol linked in several ways. The antioxidant activity exhibited by phlorotannins is mainly linked with molecular skeleton which is consist of phenol rings [24]. As the oxidative stress is tightly linked with the pathophysiological process of diabetes mellitus, phlorotannins present in marine brown algae can be used to combat oxidative stress and diabetes mellitus [25]. Several types of phlorotannins are present in different algal species, and they can be classified into six subgroups such as phlorethols, fuhalols, fucols, fucophlorethols, eckols, and carmalols based on the type of linkage between phloroglucinol units and hydroxyl groups present on the phenol rings [24]. The chemical structure of phloroglucinol and six subgroups is shown in Figure 2.

**Figure 3:**
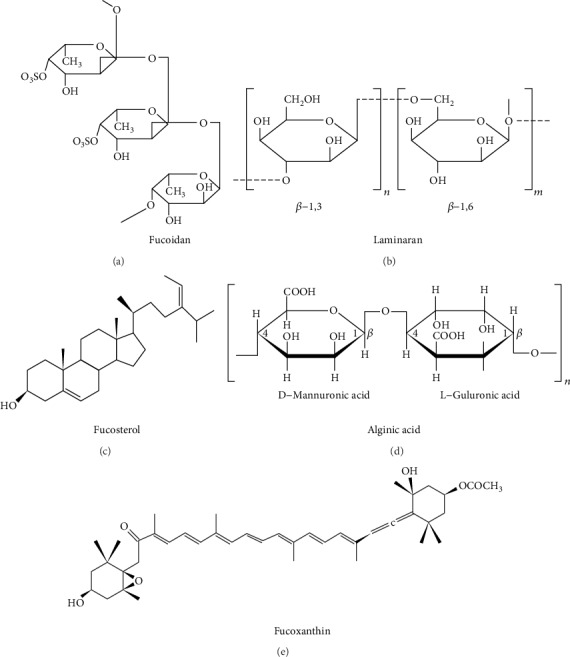
Structural unit of (a) fucoidan, (b) laminaran, (c) alginic acid, (d) fucosterol, and (e) fucoxanthin.

In addition to the phlorotannins, brown algae produce different types of polysaccharides such as fucoidans, alginates, and laminarans. Fucoidans and laminarans are water-soluble sulphated polysaccharides, whereas alginic acid is an alkali-soluble polysaccharide present in brown algae. Sulphated polysaccharides present in marine brown algae have been reported to possess various biological activities with effective therapeutic effects. Fucoidan has been reported to exhibit antioxidant, antidiabetic, and anti-inflammatory activities, whereas laminarans play a role as prebiotics and dietary fibers in addition to their antibacterial and antitumor activities. Alginic acids are mostly used in the pharmaceutical industries due to the ability to chelate the metal ions [23]. Moreover, fucosterols are the predominant sterol present in brown algae also exhibit biological properties [13]. The structure of fucoidan, laminarans, alginic acid, and fucosterols is presented in Figure 3.

Further, fucoxanthin is the accessory pigment and most abundant carotenoid present in the marine brown seaweeds and which was primarily isolated from the brown seaweeds *Fucus*, *Dictyota*, and *Laminaria.* The molecular structure of the fucoxanthin is given in Figure 3. Fucoxanthin has been reported to possess strong biological activities such as antioxidant, anticancer, and antidiabetic activities, mainly due to the unusual allenic bond and oxygenic functional group in its structure [26].

## 4. *In Vitro* and *In Vivo* Antidiabetic Potentials of Marine Brown Algae

**Figure 4:**
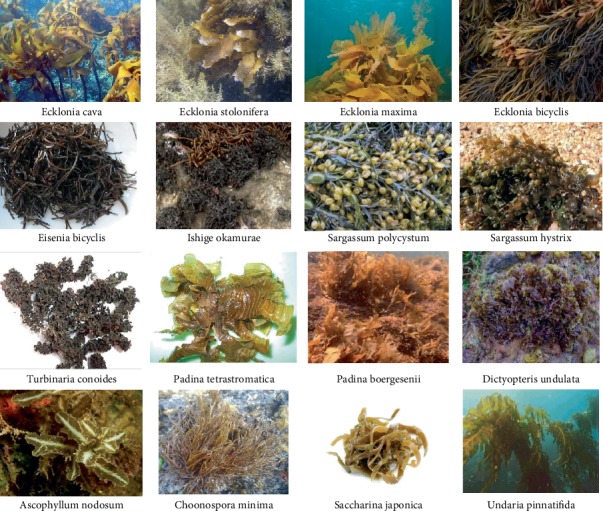
Some of the studied brown algal species for their antidiabetic effects.

The antidiabetic potential of marine macroalgae has been widely studied in the last few years due to the presence of bioactive components as mentioned above. Some of the studied brown marine algal species for their antidiabetic effects are shown in Figure 4. Among the bioactive compounds present in brown algae, phlorotannins have been identified as a potential source for the treatment of several human diseases including type 2 DM. As mentioned, the above six subgroups of phlorotannins are present in different species of brown algae, which possess significant antidiabetic action through several mechanisms [27]. In addition to the *in vitro* antidiabetic activity of marine brown algae, *in vivo* antidiabetic potential was evaluated using animal models to confirm the hypoglycaemic effect by different mechanisms [28].

### 4.1. Inhibitory Activity of *α*-Amylase and *α*-Glucosidase Enzymes

Most of the brown seaweeds which belong to the genus Ecklonia and family Lessoniaceae have been reported to exhibit antidiabetic activities mainly through the inhibitory action of *α*-amylase and *α*-glucosidase enzymes due to the presence of different phlorotannins such as eckol, dieckol, 6,6′-bieckol, phlorofucofuroeckol-A, phloroglucinol, and 7-phloroeckol [29]. The methanol extract of *Ecklonia cava* exhibited potent antidiabetic activity through the inhibitory action on *α*-glucosidase enzyme (IC_50_ -10.7 *μ*M) compared to the standard acarbose due to the presence of phlorotannin compounds, namely, dieckol, fucodiphloroethol G, 6,6′-bieckol, 7-phloroeckol, and phlorofucofuroeckol-A [29]. Similar phlorotannins were isolated from the methanol extract of *Ecklonia stolonifera* reported to possess an inhibitory activity against *α*-glucosidase enzymes which was attributed to the presence of phlorofucofuroeckol-A, dieckol, and 7-phloroeckol [29]. Furthermore, among the isolated phlorotannins, phlorofucofuroeckol-A (IC_50_: 1.37 *μ*M) and dieckol (IC_50_: 1.61 *μ*M) were found as the most effective inhibitors against the *α*-glucosidase enzyme compared to the reference drug acarbose (IC_50_: 51.65 *μ*M) [20]. The phlorotannin eckol (IC_50_: 11.16 *μ*M) isolated from *Ecklonia maxima* exhibited the potent *α*-glucosidase inhibitory activity compared to the phloroglucinol (IC_50_: 1991 *μ*M) [20]. Similarly, fucofuroeckol-A and dioxinodehydroeckol isolated from *Ecklonia bicyclis* were also reported to have an inhibitory effect on *α*-amylase and *α*-glucosidase enzymes [20]. Moreover, a significant reduction of the postprandial blood glucose level was observed by the isolated phlorotannins, dieckol, and diphlorethohydroxycarmalol in both normal and diabetes-induced mouse groups [29].

In addition to the Ecklonia species, brown seaweeds belong to the genus Eisenia also possess strong antidiabetic activity by inhibiting *α*-amylase and *α*-glucosidase enzymes [30]. Eckol, dieckol, and 7-phloroeckol isolated from *Eisenia bicyclis* also strongly inhibit the *α*-amylase enzymes by 87% at 1 mM of concentration in addition to the inhibitory activity on *α*-glucosidase enzyme and formation of advanced glycation end products [30]. Further studies confirmed that the *α*-glucosidase inhibitory activity of eckol (IC_50_: 22.78 *μ*M) is greater than the dioxinodehydroeckol (IC_50_: 34.60 *μ*M) and phloroglucinol (IC_5_: 141.18 *μ*M) whereas the *α*-amylase inhibitory activity of fucofuroeckol A (IC_50_: 42.91 *μ*M) is stronger than the dioxinodehydroeckol (IC_50_: 472.70 *μ*M) isolated from *Eisenia bicyclis* [20].

*Ishige okamurae* is a marine brown alga that belongs to the family *Ishigeaceae* and reported to have a potent antidiabetic activity through the mechanism of *α*-amylase and *α*-glucosidase inhibition. Recent studies have found that *Ishige okamurae* are rich in phlorotannins such as phloroglucinol, diphlorethohydroxycarmalol, 6-6-bieckol, octaphlorethol A, and ishophloroglucin which are responsible for the antioxidant and antidiabetic activities [28]. Among them, 6,6′-bieckol and diphlorethohydroxycarmalol showed the potent antioxidant activity which was measured by DPPH radical scavenging activity with IC_50_ values of 9.1 ± 0.4 *μ*M and 10.5 ± 0.5 *μ*M 268, respectively [28]. In addition, diphlorethohydroxycarmalol present in *Ishige okamurae* showed a potent inhibitory action against *α*-amylase (IC_50_ = 0.53 ± 0.08 mM) and *α*-glucosidase (IC_50_ = 0.16 ± 0.01 mM) to the standard drug acarbose (IC_50_ = 1.10 ± 0.07 mM and 1.05 ± 0.03 mM) [28].

Nwosu et al. [31] investigated the hypoglycaemic effect of brown algae *Ascophyllum nodosum* by means of *α*-amylase and *α*-glucosidase inhibitory effects. According to the results, *Ascophyllum nodosum* extract showed an effective inhibitory activity on *α*-amylase enzyme with an IC_50_ value of 0.1 *μ*g/ml, and further, it triggered a complete inhibition of the *α*-amylase enzyme at 2-5 *μ*g/ml. In addition, the *Ascophyllum nodosum* extract inhibited the *α*-glucosidase enzyme effectively with an IC_50_ value of 19 *μ*g/ml compared to the standard drug acarbose (IC_50_: 0.8 *μ*g/ml). Further fractionation and LC-MS analysis revealed the presence of a series of phlorotannin structures in the *Ascophyllum nodosum* extract which exhibit the inhibitory activity on *α*-amylase and *α*-glucosidase enzymes [31].

The phlorotannins present in marine brown algae *Sargassum hystrix* have been subjected to determine the hypoglycaemic effect through the inhibitory activity of carbohydrate-hydrolysing enzymes. According to the results, *Sargassum hystrix* exhibited the potent inhibitory action on *α*-amylase (IC_50_: 0.58 ± 0.01 mg/ml; IC_50 acarbose_: 0.53 ± 0.00 mg/ml; and IC_50 phloroglucinol_: 0.56 ± 0.01 mg/ml) and *α*-glucosidase (IC_50_: 0.59 ± 0.02 mg/ml; IC_50 acarbose_: 0.61 ± 0.01 mg/ml; and IC_50 phloroglucinol:_0.56 ± 0.05 mg/ml) enzymes compared to the standard acarbose and phloroglucinol [32]. Further, the antidiabetic activity of *Sargassum hystrix* has been confirmed using streptozotocin-induced rats [33]. The results revealed that *Sargassum hystrix* has reduced preprandial (186.4 mg/ml) and postprandial (186.9 mg/ml) blood glucose levels significantly in diabetic rat groups at a concentration of 300 mg/kg compared to the reference drug glibenclamide at a dose of 5 mg/kg (preprandial glucose level (195.6 mg/ml) : postprandial glucose level (104.8 mg/ml)) without any effect on the body weight of streptozotocin-induced diabetes rats [33].

Similarly, the edible seaweed *Sargassum polycystum* has been studied to evaluate the hypoglycaemic effect of streptozotocin-induced type 2 diabetic rats [34]. According to the results, ethanol (150 mg/kg) and aqueous (300 mg/kg) extracts of Sargassum *polycystum* significantly reduce the blood glucose level in diabetic rats by 27.8% and 35.2% compared to the diabetic rats treated with 250 mg/kg of metformin (84.76%) [34]. Moreover, compared to the histopathology of the pancreas in the diabetic control group, atrophy and abnormalities of the nuclei and the cytoplasm of pancreatic cells have been significantly suppressed with the treatment of ethanol (150 mg/kg), aqueous (300 mg/kg) extracts, and metformin [34]. Further, the brown seaweeds *Padina boergesenii* and *Padina tetrastromatica* have shown *in vivo* antidiabetic activities in streptozotocin-induced diabetic rats. Oral administration of the aqueous extract of *P. boergesenii* increased the activity of glycolytic enzymes and decreased the activity of gluconeogenic enzymes in diabetic rats compared to the glibenclamide treatment [35].

*Fucus vesiculosus* is a brown alga with prominent antidiabetic activities. According to the ultra-high-pressure liquid chromatography coupled to mass spectrometry (UHPLC-MS) analysis, the ethyl acetate fraction of *Fucus vesiculosus*was reported to have phlorotannins such as fucols, fucophlorethols, fuhalols, fucofurodiphlorethol, fucofurotriphlorethol, and fucofuropentaphlorethol which showed a promising antidiabetic activity through the inhibitory activity of *α*-glucosidase (IC_50_: 0.82 ± 0.05 *μ*g/ml: acarbose IC_50_: 206.6 ± 25.1 *μ*g/ml) and *α*-amylase (IC_50_: 2.8 ± 0.3 *μ*g/ml: acarbose IC_50_: 0.7 ± 0.2 *μ*g/ml) enzymes [36]. In addition to the phlorotannins, *Fucus vesiculosus* produce fucoidans and Shan et al. [37] evaluated the *α*-glucosidase inhibitory activity of fucoidan extracted from a brown alga *Fucus vesiculosus* and was reported to have the highest *α*-glucosidase inhibitory effect with IC_50_ of 67.9 *μ*g/ml compared to the standard drug acarbose (IC_50_:1000 *μ*g/ml). Significant reduction of fasting blood glucose and glycosylated haemoglobin levels were also observed in fucoidan extracted from *F*. *vesiculosus*-treated diabetes mouse group compared to the control group [37].

According to the study conducted by Chin et al. [27], the brown seaweeds, *Padina sulcata*, *Sargassum binderi*, and *Turbinaria conoides* exhibited a potent *α*-glucosidase inhibitory action in different degrees of potential. Among them, the highest inhibitory activity was reported in crude water extract of *T*. *conoides* (67.38%) at a concentration of 30 mg/ml compared to the standard acarbose (76.23%) at 1 mg/ml concentration [27]. Further, according to the study conducted in Sri Lanka, the polyphenolic rich extract of *Choonospora minima* exhibited potent antidiabetic activities through the inhibition of *α*-amylase and *α*-glucosidase enzymes [38].

### 4.2. Inhibitory Activity of Aldose Reductase (AR)

A brown alga *Ecklonia stolonifera* has been studied by Lee et al. [29], to determine the inhibitory activity of phlorotannins present in ethanol extract, hexane, dichloromethane, ethyl acetate, butanol, and aqueous fractions of *Ecklonia stolonifera*. Among them, the ethyl acetate fraction (26.63%) of *E. stolonifera* inhibited the rat lens aldose reductase more effectively more than other fractions, and further isolation revealed that the inhibitory activity on rat lens aldose reductase was mainly due to the presence of phlorotannins such a*s* 7-phloroeckol and 2-phloroeckol in ethyl acetate fraction [39]. In addition to the phlorotannins, Jung et al. [40], investigated the antidiabetic effect of fucosterol isolated from *Ecklonia stolonifera* using the inhibitory activity on rat lens aldose reductase and human recombinant aldose reductase enzymes. The results of this study revealed that fucosterols inhibited both the rat lens aldose reductase (IC_50_: 18.94 *μ*M) and human recombinant aldose reductase ((IC_50_: 18.94 *μ*M) compared to the positive control quercetin (IC_50_: 1.34 *μ*M). Hence, fucosterol isolated from *E*. *stolonifera* has ability to inhibit the intracellular accumulation of sorbitol; hence, it reduces the cataract in diabetic patients.

Laminaria japonica has undergone a taxonomic revision, and it has become *Saccharina japonica* [41]. The dichloromethane fraction of brown seaweed *Saccharina japonica* exhibited the inhibitory activity of rat lens aldose reductase enzyme due to the presence of active porphyrin derivatives such as pheophorbide-A and pheophytin-A. The result revealed that pheophorbide-A (IC_50_: 12.31 *μ*M) exhibited a potent inhibitory activity on rat lens aldose reductase compared to the pheophytin-A [41]. Further determination of chemical structure proved that the inhibitory activity of pheophorbide-A was mainly due to the presence of a carboxyl group without a phytyl group at the C-17^2^ position of the porphyrin ring. Therefore, the dichloromethane fraction of *Saccharina japonica* can be used to treat diabetic complications [42]. Further, Peng et al. [26] reported the antidiabetic potential of fucoxanthin isolated from brown seaweeds *Eisenia bicyclis* and *Undaria pinnatifida.* According to the results, fucoxanthin competitively inhibited the rat lens aldose reductase and human recombinant aldose reductase.

Moreover, Young et al. [43] studied the inhibitory activity of phlorotannins isolated from the ethyl acetate fraction of the *Eisenia bicyclis* on human recombinant aldose reductase enzyme compared to the positive control Epalrestat. Further analysis of the ethyl acetate fraction isolated the five compounds which exhibited an aldose reductase inhibitory activity, and among them, phlorofucofuroeckol-A exhibited the significant inhibition on rat lens aldose reductase (IC_50_: 6.22 *μ*M).

### 4.3. Inhibitory Activity of Angiotensin-Converting Enzymes (ACE)

A brown alga, *Ecklonia stolonifera*, has been studied for its antidiabetic effect and was reported to have important phlorotannins which exhibited an inhibitory activity on angiotensin-converting enzyme (ACE). Among the reported phlorotannins such as eckol, dieckol, and phlorofucofuroeckol-A exhibited a potent inhibitory activity on angiotensin-converting enzymes with IC_50_ values of 70.82 ± 0.25 *μ*M, 34.25 ± 3.56 *μ*M, and 12.74 ± 0.15 *μ*M. Among the isolated phlorotannins, dieckol inhibited the ACE noncompetitively. Hence, *Ecklonia stolonifera* has the potential to lower the blood pressure in diabetic patients which helps to reduce the long-term diabetic complications [44].

Paiva et al. [45] investigated the inhibitory effect of enzymatic protein hydrolysate of brown seaweed *Fucus spiralis* on ACE. Three ultrafiltrate fractions of *F. spiralis* were obtained after obtaining the protein hydrolysate with the enzymatic digestion by the mixture of two enymes cellulase and bromelain. The results revealed the potent ACE inhibitory activity in fraction 3 (IC_50_: 0.5 mg/ml) than the fraction 1 (IC_50_: 1.85 mg/ml) and fraction 2 (IC_50_: 2 mg/ml) compared to the positive control captopril (IC_50_: 0.163 ng/ml) mainly due to the amino acid composition and phlorotannins released during the enzymatic digestion [45]. Cha et al. [46] reported the ACE inhibitory activity of some Korean brown seaweeds Sargassum fusiforme, *Ishige sinicola*, *Ecklonia cava*, and *Sargassum horneri*. Among them, the highest ACE inhibitory activity was reported in 70% methanolic extract of *Sargassum fusiforme* (87%) and 70% aqueous extract of E. cava (90%) compared to others. Further, proteolytic digestion of 70% aqueous extract of E. cava revealed the presence of peptides which inhibited ACE. For further information, *Hizikia fusiforme* has undergone a taxonomic revision, and it has become *Sargassum fusiforme* [47].

In the same way, Vijayan et al. [48] found that the ethyl acetate extraction of *Sargassum wightii* exhibited the inhibitory activity on angiotensin 1-converting enzyme (ACE). During further analysis, phloroglucinol (IC_50_: 56.96 *μ*g/ml) was isolated from the ethyl acetate fraction of S. wightii showed the highest inhibitory activity on ACE compared to the reference drug captopril (IC_50_: 51.79 *μ*g/ml). According to the literature, phlorotannins which contain a low molecular weight exhibited less potency of ACE inhibition, whereas higher molecular weight phlorotannins showed higher potency of ACE inhibition. Hence, the phlorotannins with higher molecular weight such as phlorofucofuroeckol-A, 6,6′ bieckol, dieckol, and octaphlorethol A exhibited significantly higher ACE inhibition [49].

### 4.4. Inhibitory Activity of PTP 1B

Eckol, 7-phloroeckol, and phlorofucofuroeckol-A isolated from *Eisenia bicyclis*, *Ecklonia stolonifera*, and *Ecklonia* cava has the ability to inhibit protein tyrosine phosphatase 1B (PTP 1B) enzyme noncompetitively in addition to the inhibitory activity of carbohydrate-hydrolysing enzymes, aldose reductase, and formation of AGE products [50]. Moon et al. [30] evaluated the inhibitory activity of eckol, 7-phloroeckol, and phlorofucofuroeckol-A on the human recombinant PTP 1B and revealed that the isolated phlorotannins eckol, 7-phloroeckol, and phlorofucofuroeckol-A inhibited PTP 1B enzyme noncompetitively with the highest inhibitory activity of phlorofucofuroeckol-A (IC_50_: 0.56 *μ*M) compared to the 7-phloroeckol (IC_50_: 2.09 *μ*M) and eckol (IC_50_: 2.64 *μ*M). Moreover, Ezzat et al. [51] reported the fucosterol present in *E*. *bicyclis* and *E*. *stolonifera* have the ability to inhibit the PTP 1B enzyme noncompetitively.

In addition, Ali et al. [52] discovered the PTP 1B inhibitory activity of ethanol extract and fractions of brown algae *Sargassum serratifolium.* According to the results, the hexane fraction (IC_50_: 1.83 *μ*g/ml) showed the highest inhibitory activity of PTP 1B enzyme than the ethanol extract (IC_50_: 7.04 *μ*g/ml), dichloromethane (IC_50_:6.32 *μ*g/ml), ethyl acetate (IC_50_:1.88 *μ*g/ml), and butanol (IC_50_: 4.87 *μ*g/ml) fractions compared to the positive control ursolic acid (IC_50_: 1.12 *μ*g/ml). Based on the results, active hexane fraction was subjected for compound isolation and three plastoquinones such as sargahydroquinoic acid, sargachromenol, and sargaquinoic acid were identified. Among them, sargahydroquinoic acid was reported to have the highest PTP 1B inhibitory activity with IC_50_ value of 5.14 *μ*g/ml which can be a potential source for the treatment of type 2 DM [52].

Further, Feng et al. [53] isolated the 12 stigmastane-type steroids from a brown alga *Dictyopteris undulat*a and determined the hypoglycaemic potential by the inhibitory activity of PTP 1B enzyme. Among the isolated compounds, two compounds, namely, (24S)-7b-methoxy-stigmasta-5,28-diene-3b,24-diol and (24S)-7a-methoxy-stigmasta-5,28-diene-3b,24-diol, were reported to have a potent PTP 1B inhibition with the same IC_50_ value of 1.88 *μ*M compared to the positive control oleanolic acid (IC_50_: 2.78 *μ*M). Therefore, these two compounds can be further analysed to discover a new antidiabetic drug.

### 4.5. Inhibitory Activity of DPP-4 Enzyme

In 2014, Chin et al. [28] tested the antidiabetic potential of Malaysian brown seaweeds using the inhibitory activity of DPP-4 enzymes which is involved in glucose metabolism. The results revealed that the three brown seaweeds, *Padina sulcata*, *Sargassum binderi*, and *Turbinaria conoides* exhibited a potent DPP-4 inhibitory activity in a dose-dependent manner. Further fractionation revealed that the ethanolic precipitate was the most active against the DPP-4 activity. Among them, the most potent inhibitory activity against DPP-4 enzyme was observed in the ethanolic precipitate of *S*. *binderi* with IC_50_ of 2.194 mg/ml compared to the *P*. *sulcata (*IC_50_:2.306 mg/ml) and *T*. *conoides (*IC_50_: 3.594 mg/ml).

*Sargassum wightii* is a brown alga belong to the family Sargassaceae. The ethyl acetate : methanol fraction and chloroform fraction of *S*. *wightii* has been used to evaluate the antidiabetic potential through the inhibitory activity of the DPP-4 enzyme. The results indicated the highest DPP-4 inhibitory activity from ethyl acetate : methanol fraction (IC_50_: 0.013 mg/ml) more than the chloroform fraction (IC_50_: 0.028 mg/ml) compared to the reference drug diprotein-A (IC_50_: 0.007 mg/ml). In addition, the ethyl acetate : methanol (2.51 mg GAE/g) and chloroform (2.04 mg GAE/g) fractions are rich in phenolic compounds which might be attributed to the DPP-4 inhibitory activity [54].

Further studies confirmed the ability of a brown alga *Turbinaria ornata* to inhibit the DPP-4 enzyme. *T*. *ornata* is a rich source of fucoids and sulphated polysaccharides and belongs to the family Phaeophyceae. Unnikrishnan et al. [55] investigated the inhibitory activity of the DPP-4 enzyme using five solvent extracts (petroleum ether, benzene, ethyl acetate, acetone, and methanol) of *T*. *ornata*. Among the five extracts tested, the methanol extract of *T.* ornata exhibited the highest inhibitory activity (55.4%) at 80 *μ*g/ml compared to the standard diprotin A (65%). Hence, the methanol extract of *T. ornata* can be used to manage the blood glucose level in diabetes patients.

### 4.6. Inhibitory Activity of the Formation of AGEs

The phlorotannins such as phlorofucofuroeckol-A (IC_50_: 2.4 × 10^3^ *μ*M), eckol (IC_50_: 1.6 × 10^3^ *μ*M), phloroglucinol (IC_50_: 2.4 × 10^3^ *μ*M), fucofuroeckol A (IC_50_: 7.4 × 10^2^ *μ*M), dieckol (IC_50_: 7.4 × 10^2^ *μ*M), and 8,8′-bieckol (IC_50_: 6.9 × 10^2^ *μ*M) isolated from the *Ecklonia cava* have reported to exhibit the potent inhibitory effect on the formation of fluorescence bound advanced glycation end products compared to the reference drug aminoguanidine hydrochloride (IC_50_: 8.1 × 10^3^ *μ*M) [56].

Further studies on brown algae revealed the presence of phlorotannins in the methanol extract of brown algae such as *Padina pavonica*, *Sargassum polycystum*, and *Turbinaria ornata* inhibits the glucose-induced protein glycation and formation of protein-bound fluorescent advanced glycation end products (AGEs). The results revealed that the phlorotannins present in *P*. *pavonica* has a potent ability to inhibit the formation of advanced glycation end products (IC_50_: 15.16 ± 0.26 *μ*g/ml) than *S*. *polycystum* (IC_50_: 35.245 ± 2.3 *μ*g/ml) and *T*. *ornata* (IC_50_: 22.7 ± 0.3 *μ*g/ml) compared to the standard thiamine (IC_50_: 263 *μ*g/ml) and phloroglucinol (IC_50_: 222.33 *μ*g/ml) [57]. In addition, further *in vivo* studies confirmed the hypoglycaemic effect of phlorotannins (100 *μ*l) present in brown algae *P. pavonica*, *S. polycystum*, and *T*. *ornata* which exhibited the inhibitory activity on the formation of AGEs in *Caenorhabditis elegans* (a species of nematode) with induced hyperglycaemia [57].

Liu et al. [58] studied the inhibitory activity on the formation of advanced glycation end products by the phlorotannins present in acetone extract and different fractions of brown algae *Fucus vesiculosus*. The present study results indicated the higher phlorotannin content in ethyl acetate fraction (115.46 mg phloroglucinol equivalent/g) compared to the acetone extract (42.29 mg phloroglucinol equivalent/g) and other fractions. Further, subfractions of ethyl acetate fraction revealed the presence of higher phlorotannin content and (133.81 mg phloroglucinol equivalent/g) and antiglycation activity (EC_50_: 0.045 mg/ml) of subfraction 1 compared to phloroglucinol (EC_50_:0.068 mg/ml). Thus, the results explained the inhibitory activity of the formation of AGEs by the phlorotannins present in brown algae. Additionally, the present study has described the significant inhibitory activity on AGE formation by low molecular weight phlorotannins compared to the higher molecular weight phlorotannins. The summary of tested brown algae and possible active agents responsible for the antidiabetes properties are shown in Table 1.

### 4.7. Effect of Marine Brown Algae on Patients with Type 2 DM

Every new medicine and treatments started with volunteers, who have participated in clinical trials. Clinical trials are essential as they provide information for the discovery and development of new drugs. Preclinical studies provide promising data that support to conduct the relevant human studies in addition to the possible risks and early toxicity markers. Considering the results of *in vitro* and *in vivo* studies, very limited number of marine brown seaweeds have been used in the clinical trials to determine the efficacy of a particular seaweed for the development of a new drug to treat the patients with type 2 diabetes mellitus.

*Ecklonia cava* is a marine brown alga that belongs to the family Lessoniaceae which has been used to conduct the human studies as it provides strong evidence of *in vitro* and *in vivo* antidiabetic potential. A clinical trial conducted by Guzman et al. [59] found that the dieckol extract of brown alga *Ecklonia cava* has ability to reduce the postprandial blood glucose level significantly. In addition, Shin et al. [60] found that the phlorotannins extracted from *E*. *cava* exhibited strong antioxidant potential which helps to reduce the oxidative stress-induced complications of diabetes. *Ascophyllum nodosum* and *Fucus vesiculosus* are two brown algae belonging to the family Fucaceae, and Guzmán et al. [59] revealed that the ability of a commercial mixture of *Ascophyllum nodosum* and *Fucus vesiculosus* to inhibit alpha-amylase and alpha-glucosidase activity after 3 hours of ingestion. Moreover, the consumption of 500 mg and 2000 mg of brown seaweed *Fucus vesiculosus* did not affect the postprandial blood glucose and postprandial insulin levels in healthy volunteers [61].

Further clinical studies revealed the physiological effect of seaweed supplementation of the species from genus Laminaria decreased the fasting blood glucose and two hours postprandial blood glucose levels in patients with type 2 diabetes (control: 254.4 ± 22.8 mg/dl and seaweed supplementation: 203.1 ± 12.3 mg/dl) without affecting the glycated haemoglobin level [62]. *Undaria pinnatifida* is an edible brown alga belonging to the family Alariaceae. According to a clinical trial conducted by Shannon and Abu-ghannam [63] found that the daily supplementation of *Undaria pinnatifida* balanced the blood glucose levels in patients with type 2 diabetes mellitus. In addition, Sharifuddin et al. [64] found that the consumption of rice-based breakfast supplemented by *Undaria pinnatifida* reduced the postprandial blood glucose concentration in healthy volunteers.

Considering the clinical trials, unfortunately, few clinical trials were conducted so far to determine the antidiabetic potential of brown seaweed due to the lack of efficacy, lack of funding to complete the clinical trial, issues with safety, problems with sample size during recruitment and retention of patients, etc. As most of the marine brown seaweeds provide strong evidence from the preclinical trial due to the presence of phlorotannins and other bioactive compounds, researchers should be encouraged to conduct the clinical trial to search a novel drug that may be a benefit for the patients with type 2 diabetes mellitus.

## 5. Conclusion

**Figure 5:**
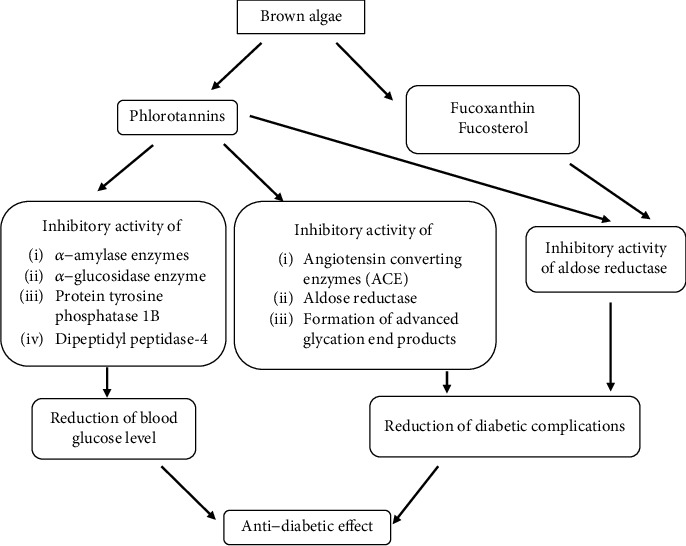
Different antidiabetic mechanisms of active agents of brown algae.

Brown algae are a rich source of bioactive compounds which exhibit significant health-promoting properties. Among them, phlorotannins have been identified in most of the studied brown algal species with most potential applications as an antidiabetic agent in addition to the fucoxanthin, fucosterol, and sulphated polysaccharides. From the available 1500-2000 brown algal species, a limited number of brown algal species have been studied worldwide to determine their antidiabetic effects. Among them, most brown algal species from family Lessoniaceae has been studied for their antidiabetic activity in addition to the brown algal species from family *Ishigeaceae*, Sargassaceae, Fucaceae, Laminariaceae, Alariaceae, and Dictyotaceae. So far, only one brown algal species from family Scytosiphonaceae has been studied in Sri Lanka to determine their antidiabetic effect as mentioned above. In this review, we have discussed about the different antidiabetic mechanisms of phlorotannins and other bioactive compounds present in marine brown algal species (Figure 5). Taken together, the search for an effective antidiabetic drug from the phlorotannins is present in brown algal benefits for the patients with type 2 diabetes. Therefore, extensive researches should be developed to utilize the marine flora more efficiently to determine the therapeutic agents that may be a benefit for the patients with type 2 diabetes.

## Figures and Tables

**Table 1:** Summary of tested brown algae and possible active agents responsible for the antidiabetes properties.

Family	Species	Active agents	Antidiabetic action
Lessoniaceae	*Eisenia bicyclis*	Dioxinodehydroeckol,	*α*-glucosidase inhibitor [20]
		7-phloroeckol	PTP 1B inhibition *α*-glucosidase inhibitor [20]
		Fucoxanthin	PTP 1B inhibition Aldose reductase inhibition Aldose reductase inhibition [26]

	*Ecklonia cava*	Dieckol	*α*-glucosidase inhibitor [43]
		Fucodiphloroethol G	*α*-amylase inhibitor[43]
		6,6′-Bieckol	PTP 1B inhibition[43]
		7-Phloroeckol	ACE inhibitor[43]
		2-phloroeckol	*α*-glucosidase inhibitor *α*-glucosidase inhibitor[43]
		Phlorofucofuroeckol-A	*α*-glucosidase inhibitor [30] PTP 1B inhibition [30] Aldose reductase inhibition [38] Aldose reductase inhibition [38] *α*-glucosidase inhibitor PTP 1B inhibition [30] ACE inhibitor AGEs inhibition [55] Aldose reductase inhibition [42]

	*Ecklonia stolonifera*	Phloroglucinol	*α*-glucosidase inhibitor [29]
		Eckol	PTP 1B inhibition *α*-glucosidase inhibitor [29]
		Dieckol	*α*-amylase inhibitor ACE inhibitor[43]
		Phlorofucofuroeckol A	PTP 1B inhibition *α*-glucosidase inhibitor [29] *α*-amylase inhibitor PTP 1B inhibition ACE inhibitor [43]
		Fucosterol	*α*-glucosidase inhibitor [29] ACE inhibitor AGEs inhibition PTP 1B inhibition Aldose reductase inhibition [39]

*Ishigeaceae*	*Ishige okamurae*	Diphlorethohydroxycarmalol	*α*-glucosidase inhibitor *α*-amylase inhibitor [28]

Sargassaceae	*Myagropsis myagroides*	Eckol	*α*-glucosidase inhibitor *α*-amylase inhibitor [29]
	*Sargassum serratifolium*	Sargahydroquinoic acid	ACE inhibitor PTP 1B inhibition PTP 1B inhibition [51]

Fucaceae	*Ascophyllum nodosum*	Methanol extract	*α*-glucosidase inhibitor [31]*α*-amylase inhibitor [31]

Laminariaceae	*Saccharina japonica*	Pheophorbide-A	Aldose reductase inhibition [41]
		Pheophytin-A	Aldose reductase inhibition [41]

*Alariaceae*	*Undaria pinnatifida*	Fucoxanthin	Aldose reductase inhibition [26]

Dictyotaceae	*Dictyopteris undulata*	(24S)-7b-methoxy-stigmasta-5, 28-diene-3b, 24-diol	PTP 1B inhibition [52]
		(24S)-7a-methoxy-stigmasta-5, 28-diene-3b, 24-diol	PTP 1B inhibition [52]
